# Supplementary material for: Interdigitated Organic Sensor in Multimodal Facemask’s Barrier Integrity and Wearer’s Respiration Monitoring
Source: Biosensors (Basel). 2022 May 6;12(5):305. doi: 10.3390/bios12050305 (PMC9138990; doi:10.3390/bios12050305)
Supplement: Supplementary file 1 [file biosensors-12-00305-s001.zip › biosensors-1687559-supplementary.pdf]

biosensors

MDPI

SUPPORTING INFORMATION

Interdigitated Organic Sensor in Multimodal Facemask's Barrier Integrity and Wearer's Respiration Monitoring

Marina Galliani [1], Laura M. Ferrari [2] and Esma Ismailova [1,*]

[1] Mines Saint-Etienne, Centre CMP, Département BEL, F-13541 Gardanne, France; marina.galliani@emse.fr
[2] INRIA, Université Côte d'Azur, 06902 Sophia Antipolis, France ; laura.ferrari@inria.fr
* Correspondence: ismailova@emse.fr

S1. PEDOT:PSS electrical properties in function of the number of printed layers.

We measured the electrical resistance of printed PEDOT:PSS patterns on the filter substrate varying the number of printed layers, from 1 to 9. The objective is to find the optimal material deposition, meaning the minimum number of layers, which enables to produce a homogeneous pattern with good electrical properties. We found 6 layers as an optimal value to minimize the material's use, enable a fast process and guarantee good conductivity.

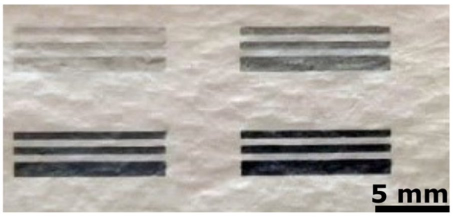

Figure S1. PEDOT:PSS-printed lines of tree different width on the facemask filter with a different number of printed layers: 1 (top left), 3 (top right), 6 (bottom left), and 9 layers (bottom right). The color change with the number of layers confirms the increasing thickness of lines.

Table S1. Resistance (ohm) of PEDOT:PSS-printed lines in function of printed layers number.

Number of layers	R (kΩ)
1	120 ± 60
3	14 ± 7
6	7 ± 4
9	2.3 ± 1.6

S2. IDE sensor design study.

Taking into account that the IDE dimension highly influences the sensor sensitivity (see section 3.2), we designed and fabricated 3 sensors to define the geometry for the e-mask prototype. The choice of an appropriate distance between the fingers (G) that is consistent with the average diameter of water drops that are deposited onto the sensor during the dampness process. Specifically, the G dimension defines the sensor's sensitivity range. The optimal W dimension was chosen to have the IDE sensitive area (shown in the circular magnified views) to cover the major portion of the mask facing the mouth. The choice of the final geometry followed the electrical properties evaluation of these sensors in respect

to the line's resistivity and detection capability towards the breathing or water droplets. The IDEs with G and W dimensions of 0.5mm and 1 mm, respectively, showed best performances.

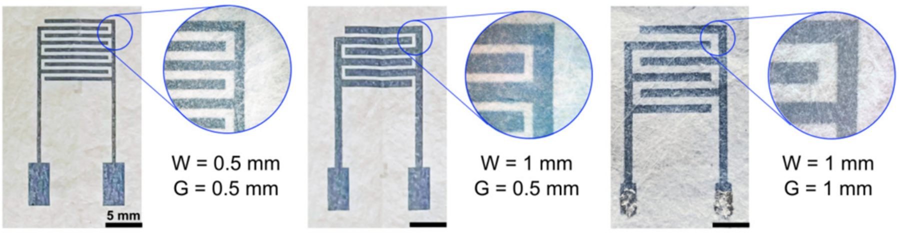

Figure S2. Printed interdigitated electrodes (IDEs) with different widths (W) and gaps (G) dimensions used for the sensor design study.

S3: Contact angle evaluation: dry and wet filter's absorbing properties study.

The contact angle measurements allowed investigating the change in the filter surface properties during the transition from a dry to a damp status. A dry facemask filter shows water-repellent properties responsible for the mask filtering capability. A deposited water drop establishes a contact angle of 136°±1.4, since the hydrophobic filter fibers do not allow the water adsorption with time. In these conditions, the mask is dry. On the other hand, a facemask filter when damp loses its water-repellent properties; water drop deposited on it is progressively absorbed, after 13 seconds, due to the capillary forces within the filter's fiber structure. This water adsorption and retention jeopardizes its protective functioning.

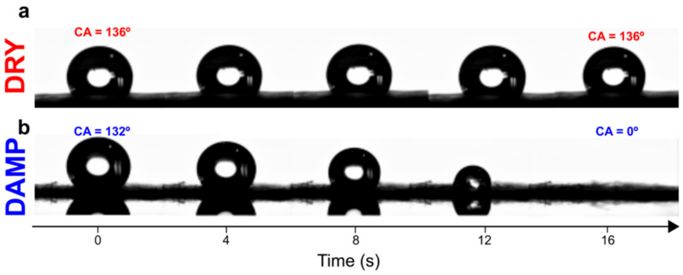

Figure S3. Contact angle measurements with time-frames photographs of water drops deposited onto a dry facemask filter (**a**) and a damp facemask filter (**b**).

S4: E-mask monitoring after removal from the wearer.

To investigate the filter's condition after e-mask removal, we looked at a particular time window of the e-mask raw signal recording. The respiration pattern is visible when the subject wears the e-mask. By the moment the subject removes the e-mask (marked by the dashed vertical line) the sensor's signal does not show the respiration pattern anymore and drops reaching a complete dry status.

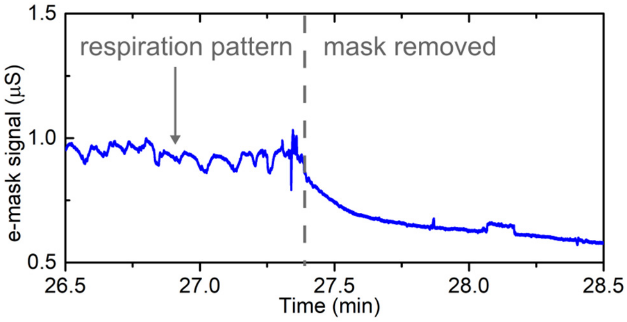

Figure S4. E-mask recording with visible respiration pattern, related to the time in which the mask is worn, and the drying process, taking place when the mask is removed.

S5: Accuracy evaluation of the respiration rate (RR) analysis.

In our work, we employed the signal peaks analysis to calculate the respiration rate. To investigate how well this method performs when the signal has noise coming from body motion, we compared the computed analysis of the recordings when the wearer is under relaxing (at rest) and running activities. The analysis provides the same accuracy in RR estimation, which is visible from the specific time window reported in the figure below. The dashed vertical line indicates when the wearer transitions from a resting position to running. The red circles result from the computed peak detection analysis for the RR calculation and denote the accurate calculation even in the presence of noise coming from motion artifacts.

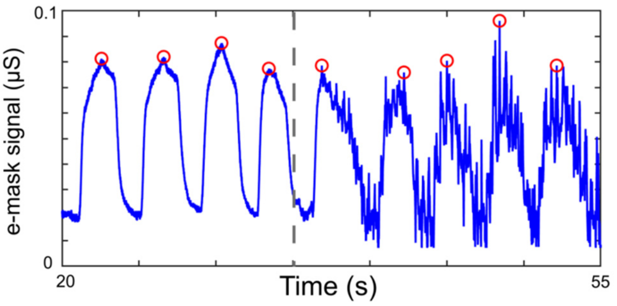

Figure S5. Time window of the e-mask raw signal recording to show the RR monitoring accuracy during relaxing and running activities.

S6: Multimodal monitoring evaluation.

The multimodal monitoring of the e-mask implies that the wetting status and RR tracking occur independently. We have evaluated and validated this by identifying the RR pattern independently from the wetting status. The sensor monitors the temporary RH changes due to respiration regardless of the wetting status, the latter reported as the decrease or increase of the output signal.

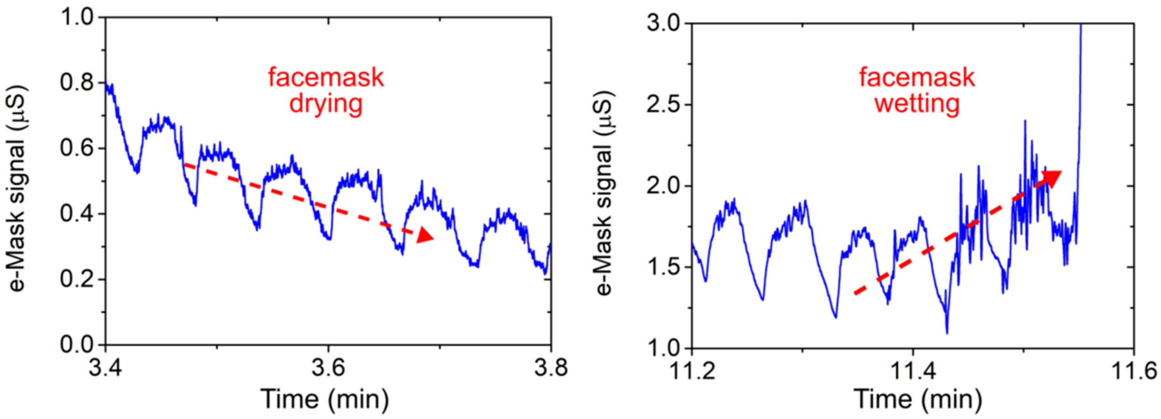

Figure S6. Time windows of the e-mask raw signal recording to show the independent and dual e-mask sensing capability. The e-mask records the respiration pattern while monitoring the water evaporation (on the left) or accumulation (on the right) from and on the mask surface respectively. These processes are indicated by a decreasing and increasing trend (red dashed lines) in the e-mask signal, respectively.

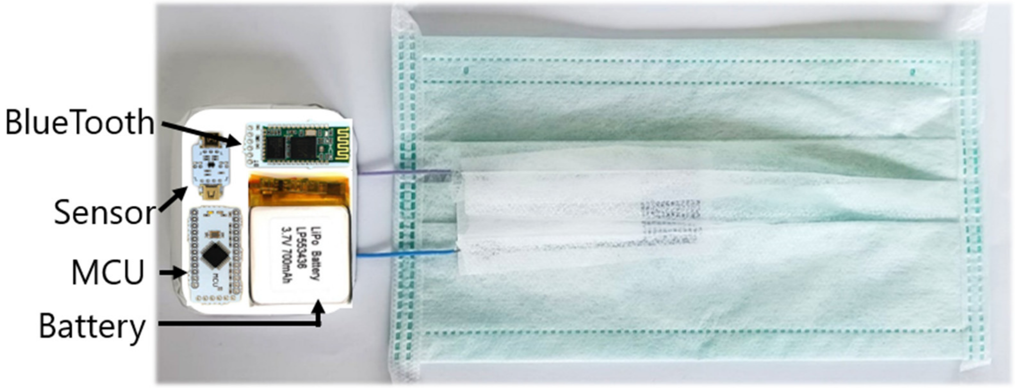

Figure S7. Picture of the E-mask with electronics modules.

The portable acquisition system is plugged to the in-mask sensor and it is composed of: a microcontroller unit (MCU), the sensor module for data acquisition, the low energy BlueTooth module for wireless data transfer, and the a battery for power supply.
